# Lymphadenectomy in Management of Invasive Bladder Cancer

**DOI:** 10.1155/2011/758189

**Published:** 2011-06-16

**Authors:** Ramy F. Youssef, Ganesh V. Raj

**Affiliations:** Department of Urology, UT Southwestern Medical Center at Dallas, J8-130 5323 Harry Hines Boulevard Dallas, TX 75390-9110, USA

## Abstract

Radical cystectomy with pelvic lymphadenectomy represents the gold standard for treatment of muscle-invasive bladder cancer. Extent of the lymph node dissection and lymph node involvement during radical cystectomy are the most powerful prognostic factors associated with poor oncological outcome. However, the optimal boundaries of the lymph node dissection during a radical cystectomy are controversial. The published literature based mostly on retrospective studies suggests that increasing the number of nodes excised may have therapeutic and diagnostic benefits without significantly increasing the surgical morbidity. These conclusions are, however, influenced by selection and surgeon biases, inconsistencies in the quality of the surgery, and node count variability. In this paper, we establish the current understanding about the utility of lymphadenectomy during a radical cystectomy for muscle-invasive bladder cancer.

## 1. Introduction

Bladder cancer (BC) is the seventh most prevalent cancer worldwide and results in significant morbidity and mortality. In the United States, BC is the fourth most common cancer in males and the ninth in females with an estimated 70,980 new cases and 14,330 deaths in 2009 [1]. Muscle-invasive BC (MIBC) accounts for virtually all the mortality from bladder cancer and represents more than a quarter (≈25–40%) of all BC. MIBC spreads from the bladder in a predictable stepwise manner to the lymph nodes and then to visceral organs. Metastatic BC is incurable and invariably fatal. For patients with MIBC, radical cystectomy (RC) with pelvic lymphadenectomy (PLND) represents the mainstay of therapy. The PLND during RC represents both a chance to accurately stage the cancer as well as a chance to remove all the cancer from the body. An extended PLND may have prognostic and therapeutic benefits. However, there is still debate regarding the boundaries of adequate PLND during RC. In this paper, we will define the state of art regarding the extent of PLND and nodal prognostic factors and their effect on oncological outcomes of BC patients.

## 2. Lymphatic Drainage of the Bladder

The anatomy of lymphatic drainage of the urinary bladder is critically important for definition of the boundaries for an adequate PLND during RC. The bladder lymphatic drainage has well-defined origin from lymphatic plexus within its wall, in the submucosa and extending into the muscles. Lymphatic channels drain through anterior, lateral, and posterior intercalated lymph nodes (LNs) located within the perivesical fat. Efferent lymphatics then drain to the external iliac, obturator, internal iliac, and presacral LNs. Lymphatic trunks leading from the pelvic LNs subsequently drain into more proximal common iliac LNs and then to aortocaval LNs [2, 3]. Skip lesions have been reported, but their rarity suggests that the pelvic LNs are the primary landing site and that metastasis occurs in an orderly progression [2, 4–8].

## 3. Surgical Boundaries of the Lymphadenectomy

**Figure 1:**
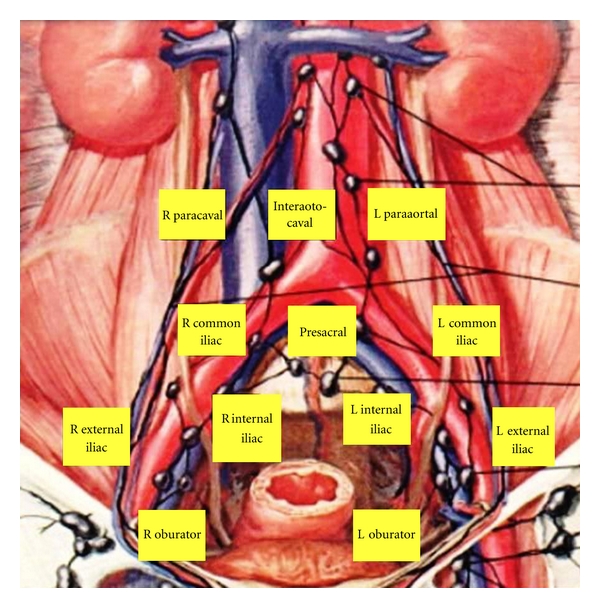
Template of extended lymph node dissection during radical cystectomy.

Figure 1 shows the template of extended PLND during RC. Extended PLND include LNs between the aortic bifurcation and common iliac vessels (proximally), the genitofemoral nerve (laterally), the circumflex iliac vein and LN of Cloquet (distally), and the internal iliac vessels (posteriorly), including the obturator fossa and the presacral LNs anterior to the sacral promontory. An extended dissection may also extend superiorly to the level of the inferior mesenteric artery. Importantly, the PLND along the external iliac vessels is completely circumferential while the proximal dissection along the common iliac and great vessels includes anterior and lateral nodal tissues [5, 7, 9]. Standard PLND differs in its cranial boundary which is limited to the level of the common iliac bifurcation. A limited PLND involves the lymph nodes in the obturator fossa.

## 4. Incidence of Lymph Node Metastasis

The incidence of positive LNs in RC specimens is between 18–30% as shown in Table 1. Predictably, the incidence of LN involvement correlates with T stage, grade, and presence of lympho vascular invasion (LVI) [5, 10, 11].  Table 2 shows the correlation with stage in the largest RC series. The incidence of LN metastasis is ≤5% for <T2, around 25% in T2, and 40–45% in T3/T4 BC.

## 5. Lymphadenectomy: Staging/Prognostic Benefits

Precise staging of LN status is an important clinico-pathological prognostic parameter following RC. Nodal involvement identifies a high-risk group that has the worst oncological outcomes and may benefit from adjuvant systemic therapies. Clearly, extending the field of PLND will increase the number of LNs removed and the chances of identifying positive nodes [5, 23, 24, 33]. Vazina et al. found 16% patients with T3/T4 disease to have LN metastasis proximal to the boundaries of standard PLND and 30% of patients with common iliac LN involvement also had involvement of presacral LNs [10]. Steven and Poulsen found 34.4% of positive LNs above the common iliac bifurcation [24]. An inadequately performed PLND may underestimate the true disease burden and underestimate the need for potentially therapeutic adjuvant therapies. An accurate PLND represents the best way to accurately stage patients with MIBC.

## 6. Lymphadenectomy: Therapeutic Benefits

Removal of involved lymph nodes theoretically can improve survival as it decreases overall tumor burden and allows the immune system and chemotherapeutics to target a smaller number of cancer cells, potentially with greater efficacy. Indeed, recent retrospective and prospective nonrandomized studies suggest a better oncological outcome from removing more LNs via an extended PLND during RC. Proponents for extended PLND note frequent involvement of nodes outside the standard PLND templates [10, 24, 34]. Some studies found more proximal LN metastases above the cranial boundary of the standard PLND (≈20%) where common iliac, presacral, and LNs above the aortic bifurcation were involved in 15–23%, 6–8%, and 4–10%, respectively [3, 5]. Additionally, up to 50% of patients with limited LN involvement can be rendered disease-free after extended PLND [2, 5]. The benefits of PLND in removing micrometastatic disease appear to be significant in BC. Extended PLND requires an additional time but does not add significantly to the morbidity of the procedure [2, 35–37]. However, there are other studies that did not find an advantage for extended PLND [6, 7, 11]. The difference between these studies may reflect the selection criteria for the patients. Further, while the removal of grossly negative but microscopically involved LN may have a therapeutic benefit, the removal of bulky involved LN is unlikely to improve survival. The level of PLND remains controversial and can only be determined by well-designed carefully controlled prospective randomized studies.

## 7. Therapeutic Benefits in Node Negative Bladder Cancer

The extent of PLND may act as a surrogate marker for overall surgical quality and survival benefits from PLND in node negative patients during RC were reported [13, 15, 31, 38, 39]. A thorough extended PLND might decrease positive surgical margins, and, hence improve the oncological outcome. The 5-year RFS with organ-confined tumors was 85% with an extended dissection compared to 64% with similar pathology undergoing a more limited PLND [31]. We recommend an extended PLND, whenever possible; even in clinically node negative patients.

## 8. Nodal Prognostic Factors

The prognosis in patients with lymph node-positive disease can be stratified by the stage of the primary bladder tumor, extent of PLND, the number of lymph nodes removed, the number of LNs involved (tumor burden), LN density, and the presence of nodal extracapsular extension. We would like to highlight the most commonly studied nodal prognostic factors.

### 8.1. Number of Lymph Nodes Removed

The median nodal counts reported from RC series is considerably variable and ranges from 9 to 30 (Table 3). This number has been used as a surrogate marker for the adequacy of PLND. A standard PLND yields an average of 8–14 nodes while extending PLND up to the aortic bifurcation often yields 25–45 nodes [2, 31, 34]. Different studies suggested different cut point for the number of LNs that should be removed to achieve adequate PLND (Table 4). SEER data showed that PLND and the number of LNs removed are variable, and dissection of at least 10 to 14 nodes during RC is the most important prognostic factor [40]. The number of LNs removed not only suggests the completeness of the PLND, but may have prognostic significance in both LN positive and negative patients [3, 13]. Koppie et al. could not find a minimum number of LNs to be sufficient for optimal oncological outcomes. Instead, the probability of survival continued to increase as the number of LNs removed increases [22].

Unfortunately, with the striking interinstitution and intrainstitution variability in node counts and variability within patients, it is hard to define an exact minimum number of LNs to be removed. The same extended PLND may yield 20 or 80 LN depending on the patient, exposure to chemotherapy, surgical technique, institution at which performed, pathologist examining the LNs, and protocol used to evaluate LNs number. Thus, a cutoff of 30 would render the same extended PLND inadequate or adequate. We believe that the extent of PLND is more important marker for better surgical quality, and, finally, oncological outcome until pathologic processing and aforementioned variables can be addressed.

### 8.2. Lymph Node Yield

Many factors might explain the variability in node yield. Clinical, anatomical, pathological, surgical, and institutional factors may play a role [2, 4]. Old age and associated comorbidities may hinder extended PLND [2, 52]. There might be some anatomic variability in the number of nodes present in different individuals [4]. Tumor stage may be associated with nodal yield [2, 4, 39]. On the other hand, negative margin status has been associated with higher nodal yield [4, 47]. Recent BCG or neoadjuvant chemotherapy might have an effect, probably by causing inflammation and fibrosis thus helping more nodal yield [4]. Sending LNs in separate packages was reported to increase the nodal yield [15, 34]. The method of pathologic evaluation including the use of fat-clearing solutions may play a role as well [2, 4]. While there were contradictory reports about the effect of surgeon volume on LN yield [4, 47], academic or teaching hospitals with a higher RC volume tend to report higher LN counts [4, 47, 53]. Recently, Fang et al. implement a policy that at least 16 LNs has to be examined pathologically. They showed that implementation of this policy can improve the survival due to increased awareness to perform a more thorough PLND [50].

### 8.3. Number of Positive Nodes (Tumor Burden)

The number of positive LNs (tumor burden) is an important prognostic factor following RC [3, 7, 13, 15, 41]. Recurrence and survival are inversely related to an increasing number of positive LNs. Some studies reported the absolute number of LNs involved as an independent prognostic factor. Others defined cutoff numbers for worse prognosis [40]. Herr et al. determined a cut-off of 4 positive LNs [39], Steven and Poulsen detrmined 5 positive LNS [24], and Stein et al. showed significantly worse survival in patients with >8 metastatic LNs [41]. Furthermore, the study from Mansoura showed a significance difference in prognosis when stratifying positive LNs (1 versus 2–5 versus 5) [11]. This was also the case in a population-based study from the SEER database (1 versus 2 versus 3 versus >3) [25]. Collectively, It is obvious that larger tumor burden in LNs is associated with poor oncological outcomes. Further, bulky positive LNs are invariably associated with a poor prognosis [29].

## 9. The Concept of “Lymph Node Density”

The LN density could be a useful prognostic concept because it combines the extent of PLND as indicated by the total number of LNs removed and the tumor burden as indicated by the number of positive nodes. Herr and Stein were the first to introduce the concept of LN density (number of positive nodes/number of removed nodes) with a cutoff of 20% to stratify outcomes [41, 42].  Table 5 describes the cut-off values suggested for LN density in different studies and the oncological outcomes based on these cut-off values. A pooled analysis of MD Anderson and Memorial Sloan-Kettering Cancer Centers showed that LN density is superior to TNM nodal status in predicting oncological outcomes after RC [36]. Nevertheless, this index can be useful only if there is a standard number of nodes that have to be removed and a standard level of PLND. Certainly, a LN density of 20% based on 1 positive LN out of 5 LNs removed is different than 20% based on 8 positive out of 40 LNs. Perhaps, this is the reason why there has not been widespread clinical use of this parameter since its introduction in 2003.

## 10. Extracapsular Extension of Lymph Node Metastasis

Extracapsular extension (perforation of the capsule of LN by tumor tissue with extranodal growth) has recently been shown to double the risk of recurrence when compared to intranodal confined LN metastasis. Fleischman et al. evaluated 101 patients who underwent RC and extended PLND for LN+ disease and analyzed the influence of extracapsular extension (found in 58% of the patients) on patient prognoses. Patients with extracapsular extension had a significantly decreased recurrence free survival (RFS) (median, 12 versus 60 months, *P* < .001) and overall survival (OS) (median, 16 versus 60 months, *P* < .001) compared with those with intranodal metastases. Multivariate analyses confirmed that extracapsular extension of LN metastases was the strongest negative predictor for RFS [19]. However, others did not find a significant association between extracapsular extension and survival after RC, leading to issue of whether this pathological finding is really of importance [55].

## 11. The Concept of “Sentinel Lymph Node”

The sentinel node is defined as the initial site of lymphatic drainage from a primary tumor. Determination of sentinel node for BC should be on individualized basis rather than on anatomic location of primary tumor due to the variability of lymphatic drainage [4]. Ghoneim and Abol-Enein introduced the concept of sentinel region (intrapelvic LNs) rather than sentinel node [6, 7]. Involvement of the intrapelvic LNs may be the first step in nodal metastasis in most BCs and skip lesions might be very rare [26, 47, 52, 55–57]. However, higher number of patients and longer followup is needed before widespread practice is accepted. 

Recently, Studer group used multimodality technique to locate the primary lymphatic landing sites of the bladder. Their technique counted upon cystoscopic-guided injection of technetium nanocolloid followed by preoperative radioactive LN detection with SPECT/CT followed by intraoperative verification with gamma probe. They found that limited pelvic PLND removed only about 50% of all primary lymphatic landing sites while extending the PLND up to the ureteroiliac crossing removed 90% [8]. Analysis of their study proves that the template or extent of PLND is more important than merely the number of LNs removed [58]. The concept of sentinel node remains investigational in BC. If sentinel nodes could be accurately identified on individual basis, this could guide the decision about the extent of PLND. Although, extended PLND can be safely and routinely performed with minimal additional morbidity, we do not currently see a need to compromise outcomes of patients with BC with a suboptimal nodal dissection and reliance on frozen section analyses.

## 12. A Tailored Approach to Lymphadenectomy

Tailoring PLND based on the clinical stage, so that patients with advanced tumors or evidence of nodal involvement would be treated with an extended PLND, whereas those with organ-confined disease and no evidence of nodal involvement would undergo standard PLND, has been advocated. However, the use of clinical stage of the primary tumor for determining of the extent of PLND is problematic [38]. Understaging could happen in approximately half of patients with clinically organ-confined disease [38, 59]. Intraoperative finding including inspection and palpation of more proximal lymphatic regions may miss a substantial percentage of positive LNs [38]. If the morbidity of PLND is minimal, then there should be minimal downside to the use of an extended PLND in all patients. Currently, there are no reliable models to guide the decision regarding the extent of PLND.

## 13. Morbidity of Lymphadenectomy

Early complications of 28% and perioperative mortality rates of 2.6–3% have been reported in large RC series [15, 26]. An extended PLND may prolong operative time by about 60 minutes. However, it does not appear to increase morbidity or mortality compared to the standard approach [2, 35–37]. Comparing LN positive versus LN negative cases, extended versus standard PLND confirmed that there are no significant differences in morbidity or mortality [2, 35–37, 41]. Extended PLND is a safe option in experienced hands that may improve oncological outcomes by decreasing positive surgical margins and resection of undetected micrometastases [36, 38]. Despite that the administration of neoadjuvant radiation or chemotherapy before RC may not increase the morbidity and mortality. Patients who have received these treatments should be judged carefully before performing an extended PLND, as there might be a higher risk of complications [9, 35, 36].

## 14. Laparoscopic-/Robotic-Assisted Surgery

Laparoscopic-/robotic-assisted RC and PLND were reported as safe feasible procedures with acceptable nodal yield and potentially equivalent oncological outcomes to open RC with no added morbidities [56, 60]. Complete removal of the LN-bearing tissue up to aortic bifurcation or inferior mesenteric artery is more challenging using minimally invasive modalities. Recently, extended PLND has been demonstrated with the robotic system, with comparable LN yields [57, 61, 62].

We believe that without long-term functional and oncologic outcome data, laparoscopic- and robotic-assisted RC should be considered investigative techniques, and patients chosen for these modalities should be appropriately selected and counseled.

## 15. Future Directions

Many of the controversies regarding the extent and the utility of PLND in RC stem from the fact that data in support or against their use have been obtained from retrospective analyses of databases and trials. As such none of these studies were powered to answer the questions regarding the utility of PLND in RC. A multicenter prospective randomized clinical trial is in the final stages of approval by SWOG (PI: Seth Lerner, Baylor College of Medicine), and it randomizes patients to standard versus extended lymphadenectomy during RC for bladder cancer. The trial is powered to detect differences in survival and when completed may truly establish the role of PLND on the outcomes of patients with bladder cancer.

## 16. Conclusions

The incidence of nodal disease in BC is around 25% and is influenced by other pathological factors, most importantly the pT stage. Extended PLND may provide prognostic and therapeutic advantages in both LN-positive and negative patients without significantly increasing morbidity. However, the extent of PLND during RC needs better definition through prospective randomized studies with long-term followup. Laparoscopic-/robotic-assisted RC and PLND are still new modalities that need longer evaluation before recommending for more patients.

## Figures and Tables

**Table 1:** Incidence of lymph node metastasis in radical cystectomy.

References	Year	No. of patients	No. of LN metastasis	(%) of LN metastasis
Vieweg et al. [12]	1999	682	193	28.1
Leissner et al. [13]	2000	447	136	30.4
Herr and Donat [14]	2001	763	193	25.3
Stein et al. [15]	2001	1,054	246	23.3
Gschwend et al. [16]	2002	686	193	28.1
Madersbacher et al. [17]	2003	507	121	23.9
Vazina et al. [10]	2004	176	43	24.4
Leissner et al. [5]	2004	290	81	27.9
Abdel-Latif et al. [11]	2004	418	110	26.3
Nishiyama et al. [18]	2004	1,113	162	14.6
Fleischmann et al. [19]	2005	507	124	24.5
Hautmann et al. [20]	2006	788	142	18.0
Shariat et al. [21]	2006	833	198	23.8
Koppie et al. [22]	2006	1,110	243	21.9
Stein et al. [23]	2007	1,621	383	23.6
Steven and Poulsen [24]	2007	336	64	19.0
Wright et al. [25]	2007	5,201	1260	24.3
Ghoneim et al. [26]	2008	2,720	555	20.4
Osawa et al. [27]	2009	435	83	19.1
Bruins et al. [28]	2009	1,600	369	23.1
Stephenson et al. [29]	2010	763	178	23.3
Seiler et al. [30]	2010	840	162	19.3

**Table 2:** Correlation of pathological T stage with LN metastases.

Study	Poulsen et al. [31]	Viewg et al. [12]	Stein et al. [15]	Madersbacher et al. [17]	Leissner et al. [5]	Vazina et al. [10]	Abdel-Latif et al. [11]	Hautmann et al. [20]	Ghoneim and Abol-Enein [32]
Year	1998	1999	2001	2003	2004	2004	2004	2006	2008
period	1990–1997	1980–1990	1971–1997	1985–2000	1999–2002	1992–2002	1997–1999	1986–2003	1971–2000
Total no. of patients	191	686	1054	507	290	176	418	788	2720

	% of LN metastasis								

pT0, pTis, and pT1	3	10	5	2	2	4	4	1	2
pT2a	18	9	18	17	13	16	7	10	8
T2b	25	23	27	34	22	40	25	41	19
pT3	51	43	45	41	44	50	48	44	39
pT4	44	41	45		50		65		36

Total	26	28	23	24	28	24	26	18	20

**Table 3:** Median number of lymph nodes removed in cystectomy series.

References	Year	No. of patients	Median number of LNs removed (range)
Stein et al. [41]	2003	244	30 (1–96)
Herr [42]	2003	162	13 (2–32)
Kassouf et al. [43]	2006	108	12 (1–58)
Kassouf et al. [44]	2008	248	12 (2–58)
Fleischmann et al. [45]	2005	101	22 (10–43)
Wright et al. [25]	2008	1260	9 (1–75)
Steven et al. [24]	2007	64	27 (11–49)
Abdel–Latif et al. [11]	2004	110	18 (mean)
Lerner et al. [46]	1993	132	31 (3–96)
Leissner et al. [13]	2000	302	15 (1–46)
Herr et al. [47]	2004	268	10 (0–54)
Koppie et al. [22]	2006	1042	9 (0–53)
Poulsen et al. [31]	1998	117	25 (9–67) extended
Dhar et al. [48]	2008	336 (Cleveland Clinic)	22 (10–43) extended
		322 (University of Bern)	

**Table 4:** Suggested number of LNs to be removed to achieve better oncological outcomes.

References	Year	No. of patients	Median number of LNs removed (range)	Cut-off number of LNs to be removed
Stein et al. [41]	2003	244	30 (1–96)	15
Konety et al. [49]	2003	361	N/A	10–14
Herr [42]	2003	162	13 (2–32)	13
Kassouf et al. [44]	2008	248	12 (2–58)	12
Fleischmann et al. [45]	2005	101	22 (10–43)	5
Wright et al. [25]	2008	1260	9 (1–75)	10
Leissner et al. [13]	2000	302	15 (1–46)	16
Herr et al. [47]	2004	268	10 (0–54)	10
Herr et al. [39]	2002	322	8 (0–44) for N0	8 for N0
			11 (1–25) for N+	11 for N+
Fang et al. [50]	2010	349	17 (0–53)	16
Dangle et al. [33]	2010	120	37 (11–87)	23–27
Shirotake et al. [51]	2010	169	10 for N0	9 for N0
			13 for N+	

**Table 5:** Cut-off lymph node densities and their effect upon oncological outcomes.

References	Year	No. of patients	Median number of LNs removed (range)	Cut-off PLND	5-Y survival rates	
					Below cut-off	Above cut-off
Stein et al. [41]	2003	244	30 (1–96)	20	17	44
Herr [42]	2003	162	13 (2–32)	20	8*	64*
Kassouf et al. [43]	2006	108	12 (1–58)	25	11	38
Kassouf et al. [44]	2008	248	12 (2–58)	20	15*	55*
Fleischmann et al. [45]	2005	101	22 (10–43)	20	15	41
Steven and Poulsen [24]	2007	64	27 (11–49)	20	25	47
Abdel–Latif et al. [11]	2004	110	18 (mean)	20	16	39
Wiesner et al. [54]	2009	152	33 (15–77)	11	8*	34*
Osawa et al. [27]	2009	435	12 (1–80)	25	12*	51*

All studies reported RFS except *reported CSS.

## Abbreviations

BC: Bladder cancer
MIBC: Muscle-invasive bladder cancer
RC: Radical cystectomy
PLND: Pelvic lymph node dissection = lymphadenectomy
LN: Lymph node
LVI: Lymphovascular invasion
RFS: Recurrence-free survival
CSS: Cancer-specific survival
OS: Overall survival
SPECT/CT: Single-photon emission computed tomography.
